# Complete Genome Sequence of *Acinetobacter calcoaceticus* CA16, a Bacterium Capable of Degrading Diesel and Lignin

**DOI:** 10.1128/genomeA.00494-17

**Published:** 2017-06-15

**Authors:** Margaret T. Ho, Brian Weselowski, Ze-Chun Yuan

**Affiliations:** a Department of Microbiology and Immunology, Western University, London, Ontario, Canada; b London Research and Development Centre, Agriculture and Agri-Food Canada, London, Ontario, Canada

## Abstract

We report here the complete assembled genome sequence of *Acinetobacter calcoaceticus* CA16, which is capable of utilizing diesel and lignin as a sole carbon source. CA16 contains a 4,110,074-bp chromosome and a 5,920-bp plasmid. The assembled sequences will help elucidate potential metabolic pathways and mechanisms responsible for CA16’s hydrocarbon degradation ability.

## GENOME ANNOUNCEMENT

The use of preexisting microbial organisms in the environment could greatly improve the efficiency of remediating industrial contaminants, such as petroleum, oil, diesel, and lignin (1–6). *Acinetobacter calcoaceticus*, a nonpathogenic Gram-negative bacterium, shows great promise in bioremediation. It was originally isolated for its ability to utilize diesel as a sole carbon source. Previous studies have shown that *A. calcoaceticus* is able to effectively degrade crude oil, diesel, pesticides, phenol, catechol, and lignin (6–11). Many species of *Acinetobacter* have been shown to secrete biosurfactants (12, 13), which further facilitate the efficiency of hydrocarbon breakdown and metabolism. This organism has the potential to be implemented in bioremediation practices and large-scale biosurfactant production. Currently, there are only two other complete assembled genomes for this species: *A. calcoaceticus* PHEA-2 (CP002177) (14) and *A. calcoaceticus* NCTC7364 (LT605059) (https://www.ncbi.nlm.nih.gov/nuccore/1160688532). Here, we provide the complete genome sequence of *A. calcoaceticus* CA16 (henceforth referenced as CA16), isolated from canola roots in southwestern Ontario.

CA16 was cultured in nutrient broth at 37°C. Genomic DNA was extracted using the GenElute bacterial genomic DNA kit by Sigma-Aldrich (catalog no. NA2120). Barcode libraries were prepared by ACGT, Inc. using fragmented genomic DNA averaging 550 bp. CA16 was sequenced on the Illumina NextSeq500 platform with 150-bp paired-end reads at 100 × genome coverage. The 10,283,145 raw reads were processed with Bcl2fastq version 1.8.4 (Illumina) and Trim Galore! (https://www.bioinformatics.babraham.ac.uk/projects/trim_galore). High-quality overlapping reads (*Q* > 30) were assembled *de novo* using SPAdes (15), which returned a 15-contig draft genome. *In silico* alignments of the draft genome were generated by Mauve (16), and missing gaps were confirmed with PCR and Sanger sequencing. Final assembly was aligned with SeqMan Pro version 12.3.1 (DNASTAR, Madison, WI, USA). Annotation was performed through the NCBI Prokaryotic Genome Annotation Pipeline.

The final assembly contains a 4,110,074-bp chromosome and a 5,920-bp plasmid. Annotation data revealed that CA16 has a G+C content of 38.69%, with a total of 3,798 coding genes, 6 rRNA operons, and 6 tRNA loci. The plasmid contains four coding sequence regions, two on each strand, and two pseudogenes. The plasmid does not carry any metabolic genes of interest, only resolvase, a Rep-B initiation protein, and DNA-binding proteins for plasmid replication. Genes involved in hydrocarbon degradation are located on the chromosome, including alkane monooxygenase (BUM88_05740, BUM88_08900), rubredoxin (BUM88_04810), esterase (BUM88_04820, BUM88_05375, BUM88_06405, BUM88_11675, BUM88_14825, BUM88_15860, BUM88_18905, BUM88_18980, BUM88_19775), and WeeF (BUM88_00230), a protein involved in biosurfactant production (12, 17–19). 

The assembled genome sequence presented here will contribute to the elucidation of regulatory pathways and metabolic networks involved with hydrocarbon degradation. This sequence will greatly facilitate future comparative genomic studies in conjunction with transcriptomics, metabolomics, and proteomics, to construct a mechanistic pathway behind CA16’s diesel and lignin degradation ability.

### Accession number(s).

The complete genome assembly project, featuring the CA16 chromosome and plasmid, has been deposited in NCBI's GenBank under the accession numbers CP020000 and CP020001. The versions described in this paper are the first versions.
