# Erratum for Ho et al., “Complete Genome Sequence of *Acinetobacter calcoaceticus* CA16, a Bacterium Capable of Degrading Diesel and Lignin”

**DOI:** 10.1128/genomeA.00808-17

**Published:** 2017-08-03

**Authors:** Margaret T. Ho, Brian Weselowski, Ze-Chun Yuan

**Affiliations:** a Department of Microbiology and Immunology, Western University, London, Ontario, Canada; b London Research and Development Centre, Agriculture and Agri-Food Canada, London, Ontario, Canada

## ERRATUM

Volume 5, no. 24, e00494-17, 2017, https://doi.org/10.1128/genomeA.00494-17. Page 1; line 30: “6 rRNA operons, and 6 tRNA loci” should read “6 rRNA gene operons, and 65 tRNA loci.”

